# Simultaneous laser excitation of backward volume and perpendicular standing spin waves in full-Heusler Co_2_FeAl_0.5_Si_0.5_ films

**DOI:** 10.1038/srep42513

**Published:** 2017-02-14

**Authors:** Zhifeng Chen, Yong Yan, Shufa Li, Xiaoguang Xu, Yong Jiang, Tianshu Lai

**Affiliations:** 1 State-Key Laboratory of Optoelectronic Materials and Technologies, School of Physics, Sun Yat-Sen University, Guangzhou, Guangdong 510275, P. R. China; 2 School of Physics and Electronic Engineering, Guangzhou University, Guangzhou, Guangdong 510006, P. R. China; 3 School of Materials Science and Engineering, University of Science and Technology Beijing, Beijing 100083, P. R. China

## Abstract

Spin-wave dynamics in full-Heusler Co_2_FeAl_0.5_Si_0.5_ films are studied using all-optical pump-probe magneto-optical polar Kerr spectroscopy. Backward volume magnetostatic spin-wave (BVMSW) mode is observed in films with thickness ranging from 20 to 100 nm besides perpendicular standing spin-wave (PSSW) mode, and found to be excited more efficiently than the PSSW mode. The field dependence of the effective Gilbert damping parameter appears especial extrinsic origin. The relationship between the lifetime and the group velocity of BVMSW mode is revealed. The frequency of BVMSW mode does not obviously depend on the film thickness, but the lifetime and the effective damping appear to do so. The simultaneous excitation of BVMSW and PSSW in Heusler alloy films as well as the characterization of their dynamic behaviors may be of interest for magnonic and spintronic applications.

Cobalt-based full-Heusler ferromagnetic alloy films have attracted much attention due to potential applications both for spintronic^1^ and magnonic^2^,^3^,^4^ devices, which can be attributed to their high Curie temperature, high spin polarization and low magnetic damping^3^,^4^,^5^,^6^,^7^. For applications of magnonic or magnon spintronic devices, spin waves are expected to serve as information carriers to realize transmission and processing of information^8^,^9^,^10^,^11^. Therefore, excitation, characterization, understanding and manipulation of spin waves become crucial. Recently, spin wave logic gates^12^ and microstructured waveguides^2^,^13^ were demonstrated. It was already reported that in cobalt-based full-Heusler ferromagnetic alloy films multiple thermal spin-wave modes (SWMs) can exist^14^,^15^. However, controllable coherent excitation of different SWMs is still difficult. Therefore, lots of observations in different systems are still necessary to clarify the excited conditions and characteristics of different SWMs, and provide more experiences for the future applications and the building of powerful theoretical models. A number of investigations are trying to fill these gaps^3^,^4^,^6^,^7^.

All-optical time-resolved magneto-optical Kerr (TR-MOKE) spectroscopy is a powerful tool to investigate spin dynamics and to get access to the local magnetic properties in time domain with the femtosecond (fs) resolution^16^,^17^. Using this method, Liu *et al*. studied the spin-wave dynamics in Co_2_MnAl^6^ and Co_2_MnSi^7^ films with in-plane external field applied, and observed only the Kittel mode spin wave (uniform precession). However, Cheng *et al*. studied the spin-wave dynamics in Co_2_FeMnAl films using TR-MOKE spectroscopy under an out-of-plane external field applied, and observed the Damon-Eshbach (DE) spin wave combined with the perpendicular standing spin wave (PSSW) modes^3^. In addition, Loong *et al*. observed the Kittel and DE modes in Co_2_FeAl_0.5_Si_0.5_ films in time domain measurement utilizing the pulsed inductive microwave magnetometry^4^.

In this work, we investigate the spin-wave dynamics in full-Heusler Co_2_FeAl_0.5_Si_0.5_ films with different thickness using TR-MOKE spectroscopy, and observe two SWMs. By analyzing the dispersion and decay characteristic, we reveal that one of the observed SWMs is the PSSW mode, while the other is the backward volume magnetostatic spin-wave (BVMSW) mode, whose coherent excitation has not been reported in Cobalt-based full-Heusler alloys yet. The BVMSW mode is excited in three films studied here with the thickness of 20, 60 and 100 nm, and done more efficiently than the PSSW mode. Moreover, apparently especial field-dependence of the spin wave lifetime and the extrinsic Gilbert damping is found. The origin is also studied and discussed. The specific combination of the dynamic observation of BVMSW and PSSW in Heusler alloy films using TR-MOKE, and the characterization of such SWMs, may be of interest for magnonic and spintronic applications.

## Results

### Magnetization dynamics and FFT spectrum

**Figure 1:**
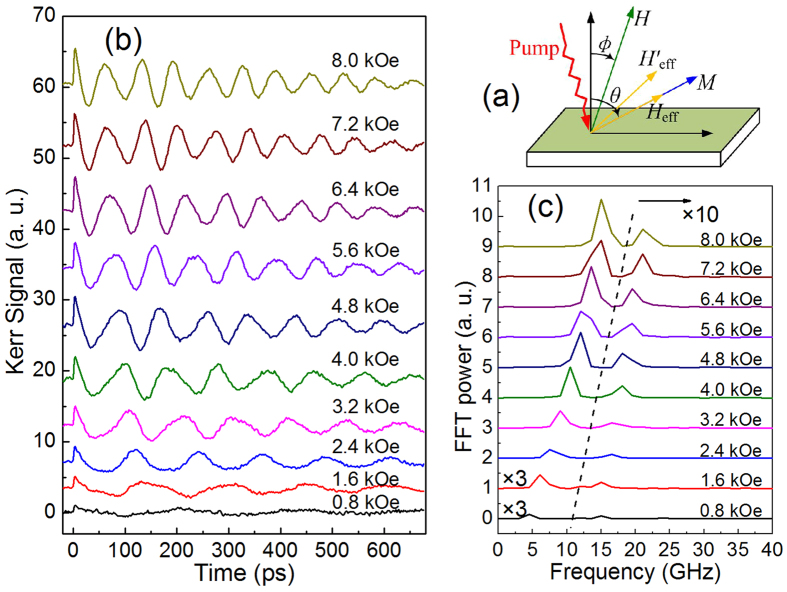
(**a**) Excitation geometry in the experiment. (**b**) Spin-wave dynamics of 60 nm thick Co_2_FeAl_0.5_Si_0.5_ film measured under a pump fluence of 12.5 mJ/cm^2^ and for different values of the external field. (**c**) FFT spectrum for the oscillatory components in (**b**). The portion of the spectrum to the right of the dashed line are amplified by 10 times.

The samples studied here are Co_2_FeAl_0.5_Si_0.5_ films with different thickness. Spin-wave dynamics are excited and measured using a TR-MOKE configuration with an out-of-plane external field applied. Figure 1(a) shows the excitation geometry. The precession of magnetization *M* is launched by the torque exerted on it as the femtosecond pumping laser transiently changes the orientation of effective field from *H*_*eff*_ to 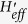
18. The details of sample preparation, measurement configuration as well as excitation mechanism, can be found in Methods section. Figure 1(b) shows the laser-induced magnetization dynamics of the 60 nm thick sample, under different DC external field (*H*) and a constant pump fluence of 12.5 mJ/cm^2^. Obvious oscillations occur in all transient traces, and show the spin wave behaviors. The large amplitude of the oscillations with respect to the demagnetization indicates the efficient excitation of spin wave. The increase of demagnetization and oscillation in amplitude with *H* is attributed to the larger out-of-plane magnetization component under higher perpendicular field. One may note that the oscillations do not simply show a damped harmonic form, implying that the pump pulses simultaneously excite more than one SWM. To identify the SWMs, the spectrum of spin waves for different *H* is obtained by extracting the oscillatory components from the magnetization dynamics and then carrying out the fast Fourier transform (FFT). The remained non-oscillatory component is an exponential decay function, and depicts the recovery of laser-induced ultrafast demagnetization.

**Figure 2:**
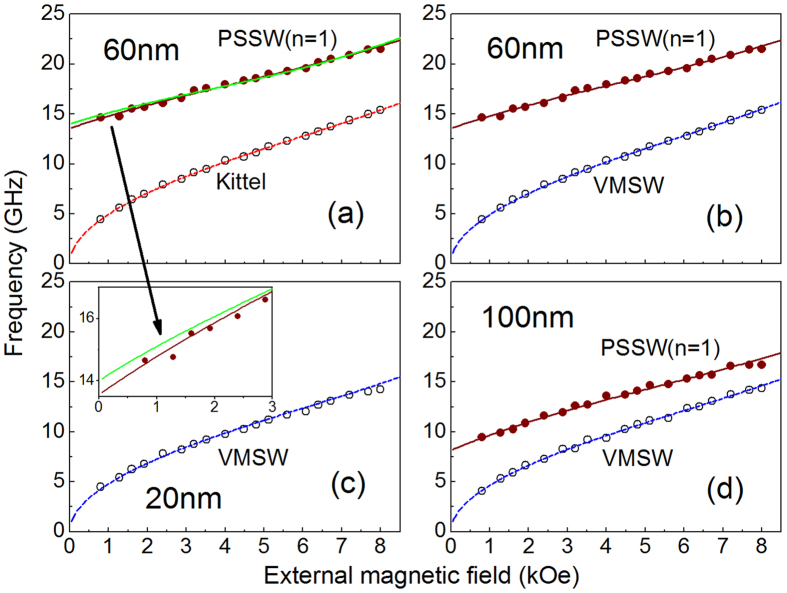
Field dependence of frequency of the HF (filled circles) and LF (open circles) modes for three films with different thickness. The wine solid lines denote the best dispersion fitting of PSSW mode, while the dashed lines represent the best dispersion fitting of Kittel and VMSW modes. The green solid line in (**a**) represents the fitting of PSSW mode using *M*_*s*_ = 741 emu/cm^3^. The inset shows the partial enlargement of (**a**).

The field-dependent FFT spectrum is plotted in Fig. 1(c). In every spectrum, two peaks occur that both shift to increasing frequency with *H*, and represent two SWMs excited. In order to simplify the description below, they are referred to as low-frequency (LF) and high-frequency (HF) modes, respectively. The strength of the LF mode is greatly stronger than that of the HF mode. The field-dependent frequency (peak position in the FFT spectrum) of the two modes is plotted in Fig. 2(a) by open and filled circles respectively, and shows the dispersion of the spin waves which can be used to identify the type or mode of spin waves.

### Dispersion analysis

According to the theory established by Kalinikos and Slavin^19^, the approximate dispersion-relation of dipolar or exchange SWMs under arbitrary effective internal magnetic field can be deduced. In our experiment, the demagnetization field and the external field applied nearly perpendicular to the film plane leads to a slant orientation of the equilibrium effective field. Thus for the volume magnetostatic spin-wave (VMSW) mode dominated by the dipole interaction, the dispersion equation (lowest order) for angular frequency can be explicitly written as


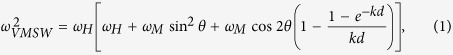


where *ω*_*H*_ = *γH* sin *ϕ*/sin *θ*, and *ω*_*M*_ = 4*πγM*_*s*_. Here *k* and *d* are the wavenumber of spin wave and the film thickness, respectively. *γ* is the gyromagnetic ratio, and *M*_*s*_ is the saturation magnetization. *θ* and *ϕ* denote the angles of the equilibrium magnetization and external field with respect to the normal of film plane, respectively, as shown in Fig. 1(a). As *k* tends to zero, the VMSW mode tends to the uniform or Kittel mode, and Eq. (1) becomes to


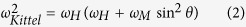


For the PSSW mode dominated by the exchange interaction, the dispersion equation is written as


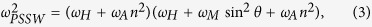


where 
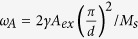
, *A*_*ex*_ is the exchange constant, and *n* denotes the order of PSSW mode. Similarly, Eq. (3) reduces to Eq. (2) as *n* = 0.

The equilibrium magnetization orientation *θ* is changed with different *H*, and meets the following equation of minimum free energy,


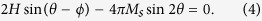


Because the frequency of the HF mode does not approach to zero with decreasing *H*, it is impossible for HF mode to be Kittel mode or VMSW mode. Considering its frequency values in the reasonable frequency range of the PSSW mode^3^,^8^, its dispersion is tried to fit with Eq. (3) plus a constraint of Eq. (4) by a least square optimization. *M*_*s*_ is fixed to the measured value of 782 emu/cm^3^ in the fitting process. The best fitting can be obtained as *n* = 1, as plotted in Fig. 2(a) by wine solid line, and agrees well with the experimental values. It gives *A*_*ex*_ = 2.83 ± 0.08 *μ*erg/cm which is comparable to the reported *A*_*ex*_ = 3.15 *μ*erg/cm of Co_2_FeSi films^20^.

For the LF mode, the frequency values seem to approach to zero with decreasing *H*. We first try to fit its dispersion using Eqs (2) and (4) with *M*_*s*_ as a fitting parameter. The best fitting is plotted in Fig. 2(a) by dash line, and gives *M*_*s*_ = 741 ± 7 emu/cm^3^. It seems to fit the experimental results well. However, we also try the best fitting with Eq. (1) instead of Eq. (2), as shown in Fig. 2(b) by dash line. It also agrees very well with the experiment frequency, giving *M*_*s*_ = 779 ± 8 emu/cm^3^ and *k* = 2.30 ± 0.15 rad/*μ*m. In comparison with the fitting by Eq. (2), this fitting gives out *M*_*s*_ closer to the measured value of 782 emu/cm^3^, while the fitting value of *k* is in the reasonable range of dipolar-interaction-dominated magnetostatic spin waves. For further showing the reasonability of *M*_*s*_ = 779 ± 8 emu/cm^3^, the fitting line of HF mode using *M*_*s*_ = 741 emu/cm^3^ is also plotted in Fig. 2(a) by green solid line, and shows worse agreement with the experimental frequency of HF mode, especially in the low field range (see the inset).

To demonstrate the effect of film thickness on the SWMs excited in the experiment, laser-induced magnetization dynamics of the samples with thickness of 20 and 100 nm are also studied. The HF mode is found to exist only in the 60 and 100 nm thick samples, while the LF mode exists in all samples. Frequency of the HF mode is significantly dependent on the film thickness but that of LF mode does not. Dispersion analysis as above is carried out. Figure 2(c and d) show the dispersion fitting of the 20 and 100 nm thick samples, respectively. For the HF mode excited in the 100 nm sample, Eqs (3 and 4) provide a good fit, giving the same *n* = 1 and *A*_*ex*_ = 3.16 ± 0.11 *μ*erg/cm. Thus, we ascertain the HF mode as the first-order PSSW mode. PSSW can be usually coherently excited in ferromagnetic films with thickness of at least few tens of nanometers^21^. The calculated PSSW frequency with 20 nm thickness and *A*_*ex*_ = 2.83 *μ*erg/cm is up to 60 GHz, much higher than the values generally reported, implying that PSSW in thinner film is difficult to be excited and measured due to higher frequency. For the LF mode excited in the two samples, their frequency dispersion data can be fit very well with Eqs (1 and 4), giving *M*_*s*_ = 787 ± 13 and 784 ± 11 emu/cm^3^, *k* = 4.88 ± 0.41 and 2.12 ± 0.26 rad/*μ*m, respectively for the samples with thickness of 20 and 100 nm. While the fittings with Eqs (2 and 4) appear to be good, but the values of *M*_*s*_ = 757 ± 10 and 731 ± 11 emu/cm^3^ given by those fitting show lager deviations from the measured value than *M*_s_ given by the fittings with Eqs (1 and 4). Based on the above comparative fitting analysis of LF mode dispersion with Eqs (1 and 2), we tend to assign the LF mode to VMSW mode. Further evidence for VMSW mode will be provided below.

### Lifetime and damping

**Figure 3:**
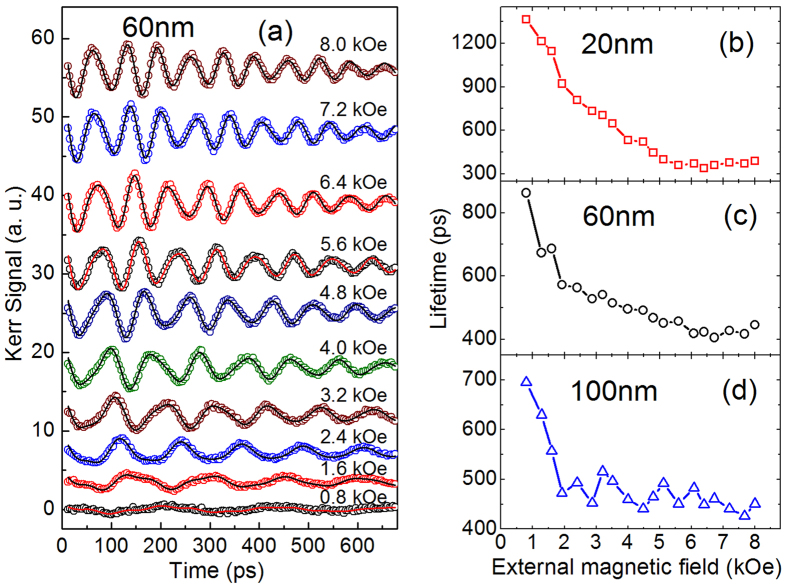
(**a**) Oscillatory components of spin-wave dynamics (open circles) for the 60 nm film shown in Fig. 1(a) and their best fittings (solid lines) with Eq. (5). (**b**–**d**) Lifetime of LF mode for the three film thicknesses obtained from fittings with Eq. (5).

The lifetime reveals the energy-dissipation rate of a spin wave and is an important parameter for magnonic applications. To achieve it, the oscillatory components in the magnetization dynamics are fitted by using the following damped harmonic sum function,


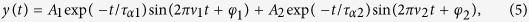


where *A*_*i*_, *τ*_*αi*_, *ν*_*i*_ and *φ*_*i*_ are the amplitude, lifetime, frequency, and initial phase of the i-th SWM, respectively. Figure 3(a) shows the best fittings (solid lines) of the oscillatory components (open circles) extracted from the magnetization dynamics of the 60 nm sample shown in Fig. 1(b). The frequency of the two modes, *ν*_1_(*H*) and *ν*_2_(*H*) given by the best fitting is almost identical to that obtained by FFT spectrum. The lifetimes *τ*_*α*1_ for three samples are plotted in Fig. 3(b–d) as functions of *H*, respectively. There is apparent difference among the three *τ*_*α*1_(*H*), especially in the low field range. All of them mainly decrease with increasing *H*, while slight increase occurs in high field range for the 20 and 60 nm samples. Maximum lifetime of 1366 ps is obtained in the 20 nm sample under *H* = 0.8 kOe. The field-dependence of lifetime is obviously distinct from the typical characteristic of Kittel mode spin wave excited under our experimental geometry^18^. For the Kittel mode, its lifetime generally does not decrease with increasing *H*.

Gilbert damping is also a vital parameter attracted much attention. For the VMSW mode, the relation between Gilbert damping factor *α* and lifetime *τ*_*α*_ is determined as ref. 11:


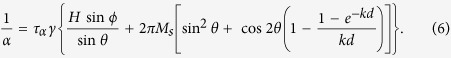


**Figure 4:**
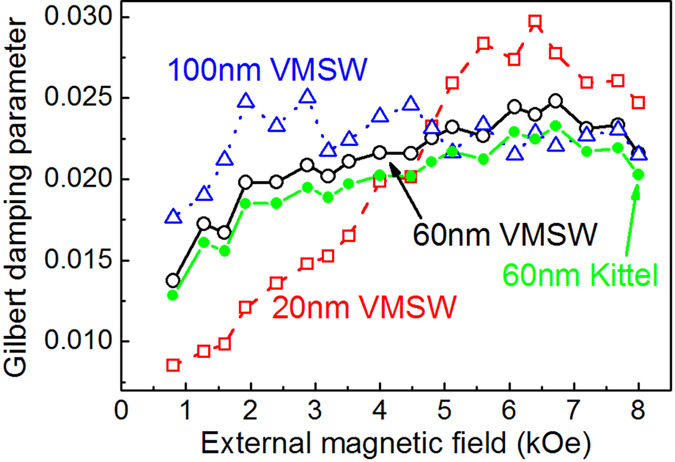
Effective Gilbert damping for the three films obtained by the damping relation for VMSW mode (open points) and Kittel mode (filled circles, 60 nm film only).

Figure 4 shows the effective Gilbert damping *α* of three samples obtained from *τ*_*α*1_ by Eq. (6). For a comparison, *α* of the 60 nm sample is also calculated using the following damping relation for Kittel mode (plotted by filled circles to distinguish)^18^:


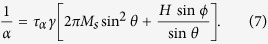


As shown in Fig. 4, the field dependence of *α* obtained by Eq. (6) (*α*_VMSW_) and (7) (*α*_Kittel_) is similar. *α*_Kittel_ remarkably increases with *H*, showing apparently extrinsic feature. Magnetic inhomogeneity is a main contribution to the extrinsic damping for Kittel mode spin-wave^18^,^21^. One of its characteristic is the competition between *H* and the distributed anisotropy field, leading to a reduction of damping with increasing *H*. Another mechanism contributed to the extrinsic damping is the two magnon scattering, which is expected to play a more remarkable role in the in-plane geometry than the perpendicular one^22^,^23^. In our experiment, because the external field *H* is applied nearly normal to the film plane, the out-of-plane angle of the equilibrium magnetization increases with increasing *H*. Thus, the possible contribution of the two magnon scattering to the extrinsic damping should decrease with increasing *H*. However, here *α*_Kittel_ obviously shows an increase with external field, implying that the extrinsic component of damping cannot be mainly from either the magnetic inhomogeneity or the two magnon scattering. That further supports that the LF mode should not be Kittel mode. In other words, LF mode should be VMSW mode. However, for VMSW mode, what is the main extrinsic origin of *α*_VMSW_? We will explore it below.

## Discussion

**Figure 5:**
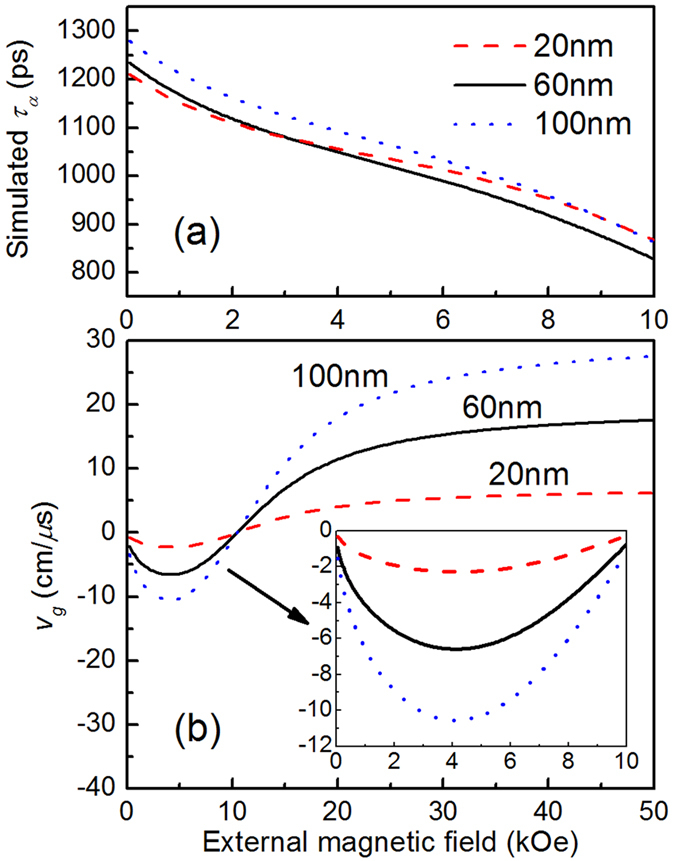
Calculated lifetime *τ*_*α*0_ (**a**) and group velocity *v*_*g*_ (**b**) for the three films as functions of external field. The inset in (**b**) shows the enlargement of *v*_*g*_ within the low field range.

Assuming an intrinsic *α*_*0*_ = 0.01 (typical value of damping for Cobalt-based full-Heusler alloys^20^), field dependent *τ*_*α*0_ for three samples are numerically calculated by Eq. (6) based on the parameters obtained from the dispersion fittings, and plotted in Fig. 5(a). One can note that the three *τ*_*α*0_(*H*) are very similar, while the slight difference comes from the slightly different *ω* for three samples. All of them decrease with increasing *H*, but the variation trend with *H* is different from the experimental one shown in Fig. 3(b–d). In the low field range, the falling slope of the calculated *τ*_*α*0_(*H*) is smaller than the experimental *τ*_*α*1_(*H*); while in higher field range the calculated one is obviously larger. Then, what results in the field dependence of *τ*_*α*1_?

VMSW mode is a propagating mode. The energy propagation along the film plane may influence the measured decay process of spin wave. Since the probing area in our experiment is located in the excited (pumping) area which can be regarded as the source of spin wave, the propagation can accelerate the decay of spin precession in the probing area^24^. Group velocity, *v*_*g*_ = *∂ω/∂k*, is just a key parameter to describe energy propagation rate. A larger |*v*_*g*_| may lead to a smaller *τ*_*α*_. Based on the parameters obtained from the dispersion fittings, *v*_*g*_ of three samples are calculated and plotted as a function of *H* in Fig. 5(b). All three *v*_*g*_ have negative values when *H* < ~10.5 kOe, implying that within this field range the group velocity is pointing in the opposite direction with the wavevector^24^,^25^, and the spin wave should be so-called BVMSW. While *H* > ~10.5 kOe, all three *v*_*g*_ have positive values, the spin wave should be so-called forward volume magnetostatic spin wave (FVMSW). Typical excitation structure for BVMSW is associated with an effective field parallel to the film plane, while for FVMSW it is done with an effective field perpendicular to the film plane. In our experiment, the out-of-plane angle of the equilibrium magnetization increases with increasing *H*. Thus, BVMSW and FVMSW can be excited possibly with different value of *H*. However, within the field range of 0–8 kOe applied in our experiment, the effective-field orientation angle *θ* is always larger than *π*/4 so that the in-plane component of effective field is dominant. Thus, BVMSW is excited preferably.

The inset in Fig. 5(b) shows the enlargement of *v*_*g*_ within *H* range of 0–10 kOe. |*v*_*g*_| of three samples present non-monotonous dependence on *H*, and reach maximums at ~4.2 kOe. Taking account for cooperative influence of intrinsic *α*_0_ and *v*_*g*_ on *τ*_*α*_, the field dependence of the experimental *τ*_*α*1_ in Fig. 3(b–d) is more easy to be understood, and can be regarded as a superimposed influence of these two factors. The calculated *τ*_*α*0_ [Fig. 5(a)] decrease with *H*, though the decreasing rates are slower than those of the experimental *τ*_*α*1_(*H*). Further taking |*v*_g_|(*H*) into account, the decreasing rates would become faster in low field. While |*v*_g_| are approaching to zero again in higher field range, *τ*_*α*1_(*H*) present slight increase. The relation between lifetime and group velocity discussed above should be another evidence for assigning the LF mode to BVMSW. Moreover, *α*_VMSW_ in Fig. 4 all initially increase with *H* for three samples, reaching a maximum and then decreasing, approximately matching the field-dependence characteristic of *v*_*g*_. That further supports the above inference. The minimum of *α*_VMSW_ is 0.0085, 0.0137, 0.0176 as *H* = 0.8 kOe for the 20, 60 and 100 nm samples, respectively. Accordingly, the intrinsic damping for each sample should be respectively smaller than these values.

In conclusion, fs-laser induced spin-wave dynamics in full-Heusler Co_2_FeAl_0.5_Si_0.5_ films are studied by employing all-optical pump-probe polar MOKE spectroscopy with an out-of-plane external field applied. Two SWMs are excited. A higher frequency mode observed in the 60 and 100 nm samples is identified to be first-order PSSW mode. The second mode with lower frequency observed in all samples is excited more efficiently and identified to be BVMSW mode whose field dependence of frequency is similar to one of Kittel mode. The Gilbert damping of BVMSW mode shows especial extrinsic feature. The relationship between lifetime and group velocity is revealed. It is found that the frequency of BVMSW mode does not obviously depend on the film thickness but the lifetime and the effective damping appear to do so. BVMSW and PSSW can be efficiently excited in our out-of-plane experimental geometry, where large-angle magnetization precession is easy to be generated. In this case, the intrinsic nonlinear of Landau-Lifshitz equation may be helpful to understand the energy transfer from pump into certain SWMs via nonlinear interaction^3^,^26^.

## Methods

The samples studied here are Co_2_FeAl_0.5_Si_0.5_ films deposited on glass substrate by magnetron sputtering in a uniform DC field at room temperature with a base pressure better than 3.0 × 10^−6^ Pa. The thickness of the samples is 20, 60 and 100 nm, respectively. The deposition rate is ~0.6 Å/s and the Ar pressure is ~0.72 Pa. All the films were annealed at 300 °C. The crystal structure of Co_2_FeAl_0.5_Si_0.5_ has been studied by grazing incidence X-ray diffraction in ref. 27. Fully ordered L2_1_, partly ordered B2, and disordered A2 structures coexist in the films. The measurement using vibration sample magnetometry (VSM) shows the in-plane magnetized feature of the samples due to the demagnetizing field, and gives the saturation magnetization of 782 ± 6 emu/cm^3^.

A time-resolved magneto-optical polar Kerr configuration is adopted to measure the spin wave dynamics. Linearly polarized laser pulse train from a Ti:sapphire regenerative amplifier with a duration of 150 fs and a repetition rate of 1 kHz at the central wavelength of 800 nm is split into pump and probe with a pump-to-probe fluence ratio larger than 30. Both the pump and probe beams are almost incident normally on the sample surface. The pump beam is focused to a spot of ~150 *μ*m in diameter, while the probe spot is located at the center of the pump spot and with diameter of approximately half that of the pump. The polar Kerr rotation of the reflected probe beam is detected by an optical balanced bridge and measured through a lock-in amplifier synchronized to an optical chopper which modulates the pump beam. The detailed description on this time-resolved Kerr setup can be found elsewhere^28^. A variable magnetic field generated by an electromagnet is applied nearly normal to the sample plane to generate larger precession angle under the laser excitation. All measurements are performed at room temperature.

The excitation geometry is shown in Fig. 1(a). The pump pulse causes the ultrafast demagnetization and transiently modulates the magnetic anisotropy, leading to the initial equilibrium effective field *H*_*eff*_ deviated to a new direction along 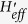. Then, a torque is exerted on the magnetization *M*, and hence launches the precession around 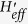
18. The length of *M* and the magnetic anisotropy recover quickly due to the spin-lattice relaxation and heat diffusion^16^, but *M* keeps on precession in a much longer time scale until its orientation returns to that of *H*_*eff*_ again.

## Additional Information

**How to cite this article:** Chen, Z. *et al*. Simultaneous laser excitation of backward volume and perpendicular standing spin waves in full-Heusler Co_2_FeAl_0.5_Si_0.5_ films. *Sci. Rep.* **7**, 42513; doi: 10.1038/srep42513 (2017).

**Publisher's note:** Springer Nature remains neutral with regard to jurisdictional claims in published maps and institutional affiliations.
